# Using self-reported data on the social determinants of health in primary care to identify cancer screening disparities: opportunities and challenges

**DOI:** 10.1186/s12875-017-0599-z

**Published:** 2017-02-28

**Authors:** A.K. Lofters, A. Schuler, M. Slater, N.N. Baxter, N. Persaud, A.D. Pinto, E. Kucharski, S. Davie, R. Nisenbaum, T. Kiran

**Affiliations:** 1 Department of Family & Community Medicine, St. Michael’s Hospital, 30 Bond Street, Toronto, ON M5B 1W8 Canada; 2 Department of Family & Community Medicine, University of Toronto, 500 University Avenue, 5th Floor, Toronto, ON M5G 1V7 Canada; 3 Centre for Urban Health Solutions, Li Ka Shing Knowledge Institute, St. Michael’s Hospital, 30 Bond Street, Toronto, ON M5B 1W8 Canada; 4 Dalla Lana School of Public Health, University of Toronto, Health Sciences Building, 155 College Street, 6th Floor, Toronto, ON M5T 3M7 Canada; 5 Department of Surgery, Li Ka Shing Knowledge Institute, St. Michael’s Hospital, 30 Bond Street, Toronto, ON M5B 1W8 Canada; 6 Cancer Care Ontario, 620 University Avenue, Toronto, ON M5G 2L7 Canada; 7 Institute for Clinical Evaluative Sciences, 2075 Bayview Avenue, Toronto, Ontario M4N 3M5 Canada

**Keywords:** Screening, Cervical cancer, Colorectal cancer, Breast cancer, Social determinants of health

## Abstract

**Background:**

Data on the social determinants of health can help primary care practices target improvement efforts, yet relevant data are rarely available. Our family practice located in Toronto, Ontario routinely collects patient-level sociodemographic data via a pilot-tested survey developed by a multi-organizational steering committee. We sought to use these data to assess the relationship between the social determinants and colorectal, cervical and breast cancer screening, and to describe the opportunities and challenges of using data on social determinants from a self-administered patient survey.

**Methods:**

Patients of the family practice eligible for at least one of the three cancer screening types, based on age and screening guidelines as of June 30, 2015 and who had answered at least one question on a socio-demographic survey were included in the study. We linked self-reported data from the sociodemographic survey conducted in the waiting room with patients’ electronic medical record data and cancer screening records. We created an individual-level income variable (low-income cut-off) that defined a poverty threshold and took household size into account. The sociodemographic characteristics of patients who were overdue for screening were compared to those who were up-to-date for screening for each cancer type using chi-squared tests.

**Results:**

We analysed data for 5766 patients for whom we had survey data. Survey participants had significantly higher screening rates (72.9, 78.7, 74.4% for colorectal, cervical and breast cancer screening respectively) than the 13, 036 patients for whom we did not have survey data (59.2, 65.3, 58.9% respectively). Foreign-born patients were significantly more likely to be up-to-date on colorectal screening than their Canadian-born peers but showed no significant differences in breast or cervical cancer screening. We found a significant association between the low-income cut-off variable and cancer screening; neighbourhood income quintile was not significantly associated with cancer screening. Housing status was also significantly associated with colorectal, cervical and breast cancer screening. There was a large amount of missing data for the low-income cut-off variable, approximately 25% across the three cohorts.

**Conclusion:**

While we were able to show that neighbourhood income might under-estimate income-related disparities in screening, individual-level income was also the most challenging variable to collect. Future work in this area should target the income disparity in cancer screening and simultaneously explore how best to collect measures of poverty.

**Electronic supplementary material:**

The online version of this article (doi:10.1186/s12875-017-0599-z) contains supplementary material, which is available to authorized users.

## Background

The social determinants of health (SDOH), such as income, race/ethnicity, education, and working and living conditions, are responsible for substantial morbidity and mortality worldwide, with income being perhaps the most important determinant [1–8]. The World Health Organization defines the SDOH as “the conditions in which people are born, grow, work, live and age, and the wider set of forces and systems shaping the conditions of daily life” [9]. The SDOH influence one’s health-related behaviours, health literacy levels, sense of self-control and self-efficacy, and access to societal resources including access to healthcare [8, 10]. The Institute of Medicine is moving to capture SDOH in electronic medical records, due to increased recognition that data on the SDOH can provide crucial information useful for treatment choices, health care system design and targeted innovations [11].

Quality primary care has been associated with an attenuation of the health effects of income inequalities [12, 13], making primary care a highly appropriate healthcare setting in which to measure and intervene on the SDOH, especially with the expansion of multidisciplinary patient-centred teams [14, 15]. However, individual-level data and practice-level data on the SDOH are rarely available within the primary care context and the best way to systematically collect and use these data in the primary care setting is not yet clear. Improving colorectal, cervical and breast cancer screening uptake for patients is an example of an area in primary care where data on the SDOH could be valuable, as disparities in cancer screening at the population level are well established, particularly related to income and foreign-born status [16–27].

In our large urban primary care practice in Ontario, Canada, we now routinely collect data on the SDOH from our patients using a self-completed survey in the waiting room. These data allow us to identify potential gaps in care related to SDOH and target our quality improvement efforts [28]. In this study, we aimed to understand: i) the relationship between the SDOH, particularly income, and colorectal, cervical and breast cancer screening, and ii) challenges and opportunities of using SDOH data from a self-administered patient survey.

## Methods

### Study setting

Our study was conducted in a large Family Health Team, a common medical home model in Ontario, Canada [29]. Our Family Health Team is comprised of six clinics, approximately 70 physicians, and 60 other health professionals serving over 35,000 patients. Our practice is situated in downtown Toronto, Canada’s most populous and sociodemographically diverse city with approximately 2.6 million people.

In the province of Ontario, cancer screening is covered through the universal provincial healthcare plan. Ontario has organized screening programs for colorectal, cervical and breast cancers, which include invitations, information about test results, and reminders for when it is time to repeat screening sent directly to residents of the province who are screen-eligible [30–32]. However, cancer screening is still fundamentally embedded in primary care.

### Collection of patient-level SDOH data

Since 2014, our practice has been routinely collecting individual-level data from our patients through a voluntary sociodemographic survey (See Additional File 1), completed in the waiting room at routine office visits. Details on survey development are published elsewhere [28]. Briefly, the survey was developed by a multi-organizational steering committee, refined by an iterative process, and pilot tested with 400 patients.

The survey is completed either on an electronic tablet or on paper and then transferred to the electronic medical record (EMR) by clerical staff. For both methods, responses to the survey questions are stored directly in the patient’s EMR and are immediately available for viewing. The survey is available in Canada’s two official languages, English and French. Participants are able to select “prefer not to answer” or “do not know” for all questions, and no questions are mandatory. As of June 30, 2015, approximately 30% of patients had participated in the survey. We used the survey to obtain individual-level data on the SDOH, specifically, immigrant status, ethnicity, household income and number of people in the household, sexual orientation, preferred language of communication, and housing status.

### Study participants

We included all patients of the family practice who were eligible for at least one of the three cancer screening types, based on age and screening guidelines [33–35] as of June 30, 2015 and who had answered at least one question on the sociodemographic survey. Adults were eligible for colorectal cancer screening if they were 50 – 74 years of age, and women were eligible for cervical and breast cancer screening if they were 21 – 69 years or 50 – 74 years respectively. We obtained data on cancer screening eligibility from a monthly cancer screening report provided to Ontario family physicians by the provincial cancer agency. The monthly report provides information to physicians on which of their patients are eligible for and due for each of the three types of cancer screening based on provincial guidelines [36]. Screening data are obtained from diagnostic and fee codes billed throughout the province. On the monthly screening report, patients who have a personal history of one of the three cancers are excluded from eligibility for that particular screening modality, and men and women with a history of a colectomy are excluded from colorectal cancer screening. Women with a history of total hysterectomy are excluded from eligibility for cervical cancer screening, and those with a history of a mastectomy or enrolment in the Ontario Breast Screening Program’s High Risk Screening Program are excluded from eligibility for breast cancer screening.

### Outcome definition

We used data provided on the provincial cancer agency’s monthly report to determine the screening status for patients eligible for colorectal and breast cancer screening. In our setting, most Pap tests were processed in a hospital laboratory and thus excluded from the provincial report, so to determine cervical cancer screening we combined data from the provincial agency with data from our practice’s EMR. Study participants were categorized as being up-to-date for screening (binary variable: yes/no) for each of the three cancers of interest. Screening status was defined as of June 30, 2015. For the three outcome variables, up-to-date was defined as at least one documented faecal occult blood test in the last two years, flexible sigmoidoscopy in the last five years, or colonoscopy in the last ten years (colorectal cancer screening), at least one Pap test in the last three years (cervical cancer screening) and at least one mammogram in the last two years (breast cancer screening).

### Survey variables

To identify individual-level income, we used responses from the sociodemographic survey to create an adapted version of the Low-Income Cutoff Before Taxes (LICO-BT) used by Statistics Canada [37]. The LICO-BT is the income threshold below which an affected family would be expected to spend at least 20% more of its household income on necessities (food, shelter, clothing) than the average family [37]. Cut-offs vary by family size and by area of residence to reflect differences in cost of living. To create an adapted LICO-BT variable, we used the responses from two survey questions: i) What was your total family income before taxes last year? (where response categories were in $30,000 increments), and ii) How many people does your income support? (see Additional file 1, questions 7 and 8). We correlated responses with the 2014 LICO-BT calculations for a Census Metropolitan Area of 500,000 inhabitants or more available from Statistics Canada. If participants reported a total family income before taxes of $0 to $29,999 and reported that their income supported two people or less they were considered below the LICO-BT. Similarly, if participants reported a total family income before taxes of $0 to $29,999 or $30,000 to $59,999 and reported their income supported between 3 to 6 people they were considered to be below the LICO-BT.

The survey includes a question on housing status with nine categories excluding “prefer not to answer” and “do not know” (see Additional file 1, question 11). We created a three-level variable for housing status (homeowners, renters, and other). Respondents in the “other” housing category reported that they lived in a boarding home, correctional facility, group home, shelter, hostel, supportive housing, on the street, or selected “other”.

The sociodemographic survey includes one question on race/ethnicity that provides sixteen potential responses, excluding “prefer not to answer” and “do not know” (see Additional file 1, question 3). Potential responses included “First Nations”, “Indigenous/Aboriginal” and “Metis”, all of which refer to Canada’s indigenous peoples. As the survey was not developed using Ownership, Control, Access and Possession principles, we chose to include data for these respondents with respondents who selected other. These principles enable self-determination over all research concerning Canada’s indigenous peoples, and include “the right to make decisions about what, why, how and by whom information is collected, as well as how it will be used and shared” [38].

### Additional study variables

Other variables collected from the EMR and monthly report included age, sex, and postal code. In addition to individual-level income, we determined neighbourhood income quintile. Patients’ postal codes were categorized into neighbourhood income quintiles using the Statistics Canada Postal Code Conversion File and data from the 2006 Census (the last Census for which accurate data are available).

### Data analysis

We used descriptive statistics to describe study participants for each variable of interest noted above, and used chi-squared tests to compare screening uptake, age, sex (for colorectal cancer screening only), and neighbourhood income quintile amongst study participants to the rest of the patient population eligible for screening for each cancer type. We compared the characteristics of patients who were overdue for screening to those who were up-to-date for screening for each cancer type using chi-squared tests. Age was characterized as both a continuous variable and a categorical variable using the following categories: 20–29 years, 30–39 years, 40–49 years, 50–59 years, 60–69 years, and 70 plus years. We compared the age of patients who were overdue for screening to those who were up-to-date for screening using both independent samples t-tests (continuous variable) and chi-squared tests (categorical variable). Participants with missing data or who had selected “prefer not to answer” or “do not know” were excluded from bivariate analyses. In bivariate analyses, ethnicity was collapsed to White versus all other ethnicities because of the small numbers in the latter category. A two-tailed test was used and a p-value of less than 0.05 was considered statistically significant. All statistical analyses were conducted using SPSS 20.0 [39].

## Results

**Fig. 1:**
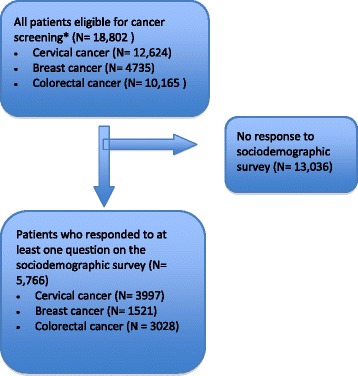
Selection of study participants

We analysed data for 5766 patients who answered at least one question on the sociodemographic survey (Fig. 1). Table 1 describes the sociodemographic characteristics of the study participants, including missing data, as well as the proportion of patients up-to-date on the three screening types by sociodemographic subgroup. These categories were not mutually exclusive; 1207 women were eligible for all three screening types. There was a large amount of missing data for the LICO-BT variable, approximately 25% across the three cohorts.

**Table 1:** Subset of patients with survey data available^a^

	Colorectal cancer		Cervical cancer		Breast cancer	
	Eligible for screening, N	Receiving Screening, row %	Eligible for screening, N	Receiving Screening, row %	Eligible for screening, N	Receiving Screening, row %
TOTAL	3028	72.9	3997	78.7	1521	74.4
Age categories						
20–29	– –	– –	618	76.7	– –	– –
30–39	– –	– –	1149	81.4	– –	– –
40–49	– –	– –	960	82.9	– –	– –
50–59	1588	67.3	760	77.9	797	72.8
60–69	1091	78.5	510	68.0	540	77.4
70+ years	349	81.1	– –	– –	184	72.8
Sex						
Male	1433	72.6	– –	– –	– –	– –
Female	1595	73.2	3997	78.7	1521	74.4
Immigrant status						
Foreign-born	1203	75.8	1604	77.7	654	76.0
Canadian-born	1722	71.4	2317	79.5	817	73.6
Prefer not to answer/Do not know/Missing	103	64.1	76	73.7	50	68.0
Ethnicity						
White-North American	1173	73.1	1325	80.1	564	74.3
White-European	724	73.5	773	78.1	334	73.4
Asian - East (e.g., Chinese, Japanese, Korean)	144	79.2	255	81.6	82	82.9
Asian - South East (e.g., Malaysian, Filipino, Vietnamese)	126	73.0	203	77.8	92	84.8
Asian - South (e.g., Indian, Pakistani, Sri Lankan)	124	74.2	299	76.6	64	78.1
Black - African (e.g., Ghanaian, Kenyan, Somali)	73	80.8	204	78.9	47	70.2
Black - Caribbean (e.g., Barbadian, Jamaican)	87	75.9	169	77.5	59	72.9
Black - North American (e.g., Canadian, American)	17	64.7	29	72.4	8	75.0
Indian - Caribbean (e.g., Guyanese with origins in India)	40	57.5	43	81.4	24	70.8
Latin American (e.g., Argentinean, Chilean, Salvadorian)	86	73.3	129	76.7	35	80.0
Middle Eastern (e.g., Egyptian, Iranian, Lebanese)	40	70.0	67	68.7	13	84.6
Mixed heritage (e.g., Black- African and White-North American	25	64.0	93	76.3	11	72.7
Other (including First Nations/Indigenous/Aboriginal not included elsewhere/Inuit/Métis)	113	69.0	154	84.4	55	56.4
Prefer not to answer/Do not know/Missing	256	68.8	254	74.8	133	71.4
Neighbourhood income quintile						
Q1	714	73.2	949	76.4	337	70.0
Q2	447	72.9	633	79.5	230	73.0
Q3	451	71.8	612	78.1	251	74.1
Q4	477	73.2	600	79.0	261	75.9
Q5	738	75.5	826	82.1	353	77.6
Missing	201	63.7	377	75.9	89	78.7
Low-income cut-off, before taxes (LICO-BT)^b^						
Below low-income cut-off	710	68.6	836	76.4	312	64.7
Above low-income cut-off	1529	74.9	2130	80.9	769	79.8
Prefer not to answer/Do not know/Missing	789	72.9	1031	75.6	440	71.8
Sexual orientation						
Heterosexual	2305	72.8	3395	79.3	1300	74.6
Gay/Bisexual/Lesbian/Queer/Two-Spirit/Other	463	72.4	277	75.1	71	66.2
Prefer not to answer/Do not know/Missing	260	74.6	325	75.1	150	76.7
Language						
What language would you feel most comfortable speaking in with your healthcare provider?						
English	2875	72.9	3732	78.6	1437	74.4
Other Language	136	72.1	252	79.4	77	77.9
Prefer not to answer/Do not know/Missing	17	76.5	13	76.9	7	42.9
In what language would you prefer to read healthcare information?						
English	2750	73.1	3656	78.7	1379	74.3
Other language	115	67.8	211	79.1	61	73.8
Prefer not to answer/Do not know/Missing	163	72.4	130	76.9	81	77.8
Housing						
Own Home	1508	75.5	1744	81.9	805	79.0
Renting Home	942	71.3	1697	76.6	442	73.8
Other	325	65.5	284	74.3	147	59.2
Prefer not to answer/Do not know/Missing	253	72.3	272	75.0	127	65.4

^a^Screening rates are reported as row percentages

^b^LICO-BT derived from 2014 Statistics Canada LICO-BT calculations with the following modifications:

i) if participants reported a total family income before taxes of $0 to $29,999 (question #7) and reported that their income supported ≤ 2 people (question #8) they were below the LICO-BT

ii) if participants reported a total family income before taxes of $0 to $29,999 or $30,000 to $59,999 (question #7) and reported their income supported between 3 to 6 people (question #8) they were below the LICO-BT

iii) if participants reported a total family income before taxes of $0 to $29,999 or $30,000 to $59,999 or $60,000 to $89,999 (question #7) and reported their income supported seven or more people (question #8) they were below the LICO-BT

**Fig. 2:**
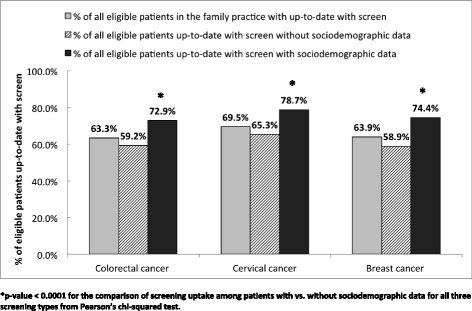
Colorectal, cervical, and breast cancer screening rates for all eligible patients and eligible patients with survey data. *p-value < 0.0001 for the comparison of screening uptake among patients with vs. without sociodemographic data for all three screening types from Pearson’s chi-squared test

Patients who completed the sociodemographic survey were significantly more likely to be up-to-date on colorectal, cervical and breast cancer screening than those who did not complete the sociodemographic survey (Fig. 2). Study participants were most likely to be up-to-date on cervical cancer screening (78.7%) and least likely for CRC screening (72.9%), which also held true for the general practice population. No significant differences were observed for any of the screening modalities based on age or neighbourhood income quintile (data not shown) between participants who completed the sociodemographic survey and those who did not complete the sociodemographic survey. Among those eligible for colorectal cancer screening, female patients were more likely to complete the sociodemographic survey than male patients.

In bivariate analyses (Table 2), age, immigrant status, income and housing showed statistically significant associations with cancer screening. Patients who were up-to-date with colorectal cancer screening were significantly older than those were overdue, whereas those who were up-to-date with cervical cancer screening were significantly younger than those were overdue. Foreign-born patients were more likely to be up-to-date on colorectal screening than their Canadian-born peers but showed no significant differences in breast or cervical cancer screening. Although there were no significant differences for any of the screening modalities based on neighbourhood income quintile, the derived LICO-BT variable was associated with all three screening types. People living below the LICO-BT were less likely to be up-to-date on screening for all screening types and this difference was most marked for breast cancer screening. Housing status was also associated with all three cancer screening types. Screening was less common among patients who rented a home and those who reported their housing as “other” than among those who owned a home. There were differences in breast and cervical cancer screening between heterosexual and non-heterosexual women but these were not statistically significant.

**Table 2:** Bivariate results for screening rates among patients with survey data^a^

	Colorectal cancer			Cervical cancer			Breast cancer		
	Overdue for colorectal screening, %	Up-to-date for colorectal screening, %	*p*-value	Overdue for cervical cancer screening, %	Up-to-date for cervical cancer screening, %	*p*-value	Overdue for breast cancer screening, %	Up-to-date for breast cancer screening, %	*p*-value
TOTAL	821	2207		853	3144		389	1132	
Age categories									
20–29	– –	– –	**<0.0001**	16.9	15.1	**<0.0001**	– –	– –	0.141
30–39	– –	– –		25.1	29.7		– –	– –	
40–49	– –	– –		19.2	25.3		– –	– –	
50–59	63.3	48.4		19.7	18.8		55.8	51.2	
60–69	28.6	38.8		19.1	11.0		31.4	36.9	
70+ years	8.0	12.8		– –	– –		12.9	11.8	
Age, mean (SD)	57.8(6.7)	60.6(6.8)	**<0.0001**	44.5(13.8)	42.7(12.1)	**<0.0001**	59.3(7.3)	60.1(6.7)	0.06
Sex									
Male	47.9	47.1	0.715	– –	– –	– –	– –	– –	– –
Female	52.1	52.9		100.0	100.0		100.0	100.0	
Immigrant status									
Foreign-born	37.1	42.6	**0.008**	43.0	40.3	0.171	42.1	45.3	0.287
Canadian-born	62.9	57.4		57.0	59.7		57.9	54.7	
Ethnicity									
White	68.6	68.4	0.934	54.9	56.4	0.455	66.7	64.0	0.372
Other ethnicity	31.4	31.6		45.1	43.6		33.3	36.0	
Neighbourhood income quintile									
Q1	23.3	23.7	0.692	26.3	23.1	0.062	26.0	20.8	0.219
Q2	14.7	14.8		15.2	16.0		15.9	14.8	
Q3	15.5	14.7		15.7	15.2		16.7	16.4	
Q4	15.6	15.8		14.8	15.1		16.2	17.5	
Q5	22.0	25.2		17.4	21.6		20.3	24.2	
Low-income cut-off, before taxes (LICO-BT)									
Below low-income cut-off	36.7	29.8	**0.002**	32.7	27.0	**0.006**	41.5	24.8	**<0.0001**
Above low-income cut-off	63.3	70.2		67.3	73.0		58.5	75.2	
Sexual orientation									
Heterosexual	83.0	83.4	0.845	91.1	92.8	0.099	93.2	95.4	0.115
Gay/Bisexual/Lesbian/Queer/Two-Spirit/Other	17.0	16.6		8.9	7.2		6.8	4.6	
What language would you feel most comfortable speaking in with your healthcare provider?									
English	95.3	95.5	0.828	93.9	93.6	0.779	95.6	94.7	0.488
Other Language	4.7	4.5		6.1	6.4		4.4	5.3	
Housing									
Own Home	49.1	56.3	**<0.0001**	40.1	48.6	**<0.0001**	49.0	60.6	**<0.0001**
Renting Home	36.0	33.2		50.6	44.2		33.6	31.1	
Other	14.9	10.5		9.3	7.2		17.4	8.3	

^a^Screening rates reported as column percentages

Statistically significant *p*-values were bolded

We did not conduct multivariable analyses because of the large amount of missing data for income (approximately 25%), which was our main variable of interest. It was not possible to use neighbourhood income as a proxy in multivariable analysis because there was not a high level of agreement between neighbourhood income and the LICO. For example, 50.9% of participants in the lowest income quintile and 65.5% of participants in the second lowest income quintile lived above the LICO.

To better understand our results, we looked at the relationship between housing status and LICO-BT status. Of those who were homeowners, the vast majority lived above the LICO-BT (90.1% for the CRC screening cohort, 90.9% for cervical cohort, 89.4% for breast cohort). Similarly, of those who lived in “other” housing i.e. were neither homeowners nor renters, most were below the LICO-BT (81.1% for CRC cohort, 76.2% for cervical cohort, 76.6% for breast cohort).

## Discussion

We used self-reported SDOH data within the primary care setting to describe the association between the SDOH and clinical outcomes: colorectal, cervical and breast cancer screening. We found that participants living below a low-income threshold indicative of poverty and people who did not own their homes were significantly less likely to be screened for cancers than their more advantaged peers. However, we also found that patients who responded to the sociodemographic survey were not typical of all patients in the practice, with higher cancer screening rates than patients who did not respond to the survey. Although we were able to use survey data to formulate a low-income cut-off based on the Statistics Canada definition, there was a large amount of missing income information, approximately 25% across the three cancer screening cohorts, precluding us from conducting multivariable analyses. It is possible that income was on the causal pathway of the relationship we observed between housing status and screening. Other variables (e.g. age) may have also confounded the relationships we observed between income and screening and housing and screening or acted as intermediate factors.

Our study highlights opportunities in the use of self-reported SDOH data in primary care. We found a significant association between the low-income cut-off variable and cancer screening despite the high amount of missing income data, and despite neighbourhood income quintile not being associated with cancer screening. Most studies on income inequities in health use neighbourhood income as a proxy for individual income, as the latter is often not available [19, 23, 25, 27, 40–44]. Our results demonstrate that neighbourhood income may under-represent income-related inequalities and were not in high agreement with the individual-level LICO. This may be especially true in urban centres like Toronto where the demographics of neighbourhoods are changing rapidly and faster than a quinquennial census can accurately track [45].

We also found intriguing results based on housing status. We found no other studies that examined the relationship between housing status and cancer screening. However, housing status has been associated with other aspects of cancer care in the international literature. A recent U.S. study showed that both renters and people in unstable housing had delays in the time to diagnostic resolution after a cancer screening abnormality when compared to homeowners, potentially due to barriers such as coordination and scheduling, transportation, childcare and low literacy [46]. Hagedoorn et al. found that home ownership (versus tenancy) provided a beneficial effect on lung cancer mortality in Belgium [47]. Housing status may simply be a marker of income, but it may also be a marker of wealth or social stability. Future research should further explore the causal pathway that lies between housing and cancer screening, taking into account sociodemographic variables that have been previously found to be associated with cancer screening, such as age, income, immigrant status, and co-morbidities, and that could serve as confounders for observed associations [19, 23, 26, 27, 48, 49].

Our results also highlight several challenges in collecting individual-level income status using self-reported data. One-quarter of participating patients did not provide a household income; missing data for other social determinants were otherwise less than 10%. Our findings of missing income are in line with previous literature. Kirst et al. found that 67% of Ontario residents were uncomfortable with disclosing household income and there was a general lack of awareness of the importance of collecting sociodemographic data [50]. Similarly, a national survey conducted in 2009 found that 65% of Canadian respondents were uncomfortable with the collection of household income in healthcare settings [51]. Respondents were most comfortable with sociodemographic data being directly collected by their family physician and certain groups were more comfortable with the collection of income, such as visible minorities and males [51].

It is possible that asking about socioeconomic status in different ways might have been more acceptable to our survey participants and might have led to less missing data. The question “Do you have difficulty making ends meet at the end of the month?” has been found to be a good predictor in a Canadian primary care population of being below the low-income cut-off, with a sensitivity of 98% and specificity of 40%, with the caveat that non-English speakers may not understand this colloquial term [52]. Future research could test such questions for their acceptability and validity in our setting [15]. Interestingly, in the pilot testing of our sociodemographic survey, only 10.1% of patients did not answer the income question [28], stressing the importance of ongoing post-implementation surveillance of such initiatives.

We also experienced several other challenges with collecting and using SDOH data. First, although there were a large number of categories for ethnicity in the survey, many of these categories had quite small cell sizes. We also had data on indigenous persons that we did not feel comfortable using due to a lack of adherence to Ownership, Control, Access and Possession principles [38]. We ultimately collapsed these categories into White vs. non-White for bivariate analyses. However, different categorizations might have led to more meaningful ethnicity-based screening results. How to best categorize ethnicity while maximizing both sample size and granularity needs to be further explored. Second, our survey was only available in English or French, which may explain the low proportion of patients in our survey who reported their preferred language as one other than English. This may have dissuaded some foreign-born patients from participating in the survey, and may partially explain why we saw no disparities based on immigrant status. Third, study participants were not representative of all patients in the practice, as noted by their higher screening rates. Participants were all willing to answer a waiting room survey, so it is plausible that they may have been more likely to come in for visits than other patients and that they may also have been more likely to be intrinsically motivated to participate in prevention and screening manoeuvres. These latter two points may partially explain why we found no disparities in screening for foreign-born patients despite well-documented disparities at the provincial level [23, 26, 44]. Fourth, although a sizeable proportion of our patients have completed the survey, detecting differences in care among some patient groups (e.g. non-heterosexual patients, certain ethnic groups) would likely require a larger sample. Fifth, the survey did not include a question on education, another known social determinant of health. Prior research demonstrates that higher education levels are positively associated with colorectal, cervical cancer, and breast cancer screening adherence [53–55]. Sixth, we were unable to track family history of cancer, which has been shown to be a motivator for cancer screening [56–59]. Seventh, younger participants in our study had less opportunity for cancer screening, which may at least partially explain our findings on age and colorectal cancer screening. Finally, it is plausible that the missing data encountered in all questions was non-random and potentially biased our findings.

## Conclusions

Our experiences highlight key considerations for primary care providers and researchers interested in collecting and intervening on SDOH in primary care. While we were able to show that neighbourhood income might under-estimate income-related disparities in screening, individual-level income was also the most challenging variable to collect. Future work in this area should focus on targeted interventions that reduce income-related disparities in cancer screening [60] and simultaneously continue to explore how best to collect socioeconomic data, particularly measures of poverty. Further research is also needed to understand whether housing is an independent risk factor for under-screening.

## Additional file

Additional file 1: Sociodemographic survey. Sociodemographic survey completed in the waiting room at routine office visits. (PDF 220 kb)

## Abbreviations

EMR: Electronic medical record
LICO-BT: Low-Income Cutoff Before Taxes
SDOH: Social determinants of health
