# Supplementary material for: Using self-reported data on the social determinants of health in primary care to identify cancer screening disparities: opportunities and challenges
Source: BMC Fam Pract. 2017 Feb 28;18:31. doi: 10.1186/s12875-017-0599-z (PMC5330155; doi:10.1186/s12875-017-0599-z)
Supplement: Additional file 1: — Sociodemographic survey. Sociodemographic survey completed in the waiting room at routine office visits. (PDF 220 kb) [file 12875_2017_599_MOESM1_ESM.pdf]

Additional file 1. Sociodemographic survey

Measuring Health Equity

Please tell us about yourself.

We want to ask you 11 brief questions as part of our ongoing work to improve access, quality of care for all patients and identify health inequities. It should take approximately 2-5 minutes to complete.

Your participation is VOLUNTARY and you can stop at any time.

You do not have to complete the survey if you don't want to. You can skip questions.

The information you share with us will be safely kept with your medical file.

This will not affect your access to care.

PLEASE NOTE SURVEY IS DOUBLE-SIDED

1. What language would you feel most comfortable speaking in with your healthcare provider? Choose **ONE**.

Choose one	Greek	Punjabi	Vietnamese
English	Hebrew	Russian	Other
Amharic	Hindi	Serbian	Prefer not to answer
Arabic	Hungarian	Slovak	Do not know
ASL	Inuktitut	Somali	Prefer not to answer
Bengali	Italian	Spanish	Do not know
Chinese (Cantonese)	Karen	Tagalog	
Chinese (Mandarin)	Korean	Tamil	
Cree	Nepali	Tigrinya	
Czech	Ojibwe	Turkish	
Dari	Oji-Cree	Twi	
Farsi	Polish	Ukrainian	
French	Portuguese	Urdu	

2. Were you born in Canada?

☐ Yes

☐ No

☐ Prefer not to answer

☐ Do not know

If no, what year did you arrive in Canada? ______________

3. Which of the following best describes your racial or ethnic group? Choose **ONE**.

☐ Asian – East (e.g. Chinese, Japanese, Korean)

☐ Asian – South (e.g. Indian, Pakistani, Sri Lankan)

☐ Asian – South East (e.g. Malaysian, Filipino, Vietnamese)

☐ Black – African (e.g. Ghanaian, Kenyan, Somali)

☐ Black – Caribbean (e.g. Barbadian, Jamaican)

☐ Black – North American (e.g. Canadian, American)

☐ First Nations

☐ Indian – Caribbean (e.g. Guyanese with origins in India)

☐ Indigenous/Aboriginal not included elsewhere

☐ Inuit

☐ Latin American (e.g. Argentinean, Chilean, Salvadorian)

☐ Métis

☐ Middle Eastern (e.g. Egyptian, Iranian, Lebanese)

☐ White – European (e.g. English, Italian, Portuguese, Russian)

☐ White – North American (e.g. American, Canadian)

☐ Mixed Heritage (e.g. Black – African and White-North American)

☐ Other (s)

☐ Prefer not to answer

☐ Do not know

4. Do you have any of the following? (Circle **ALL** that apply)

None | Chronic illness | Developmental Disability | Learning disability

Mental illness | Physical disability | Sensory disability (i.e. hearing or vision loss)

Drug or alcohol dependence | Other | Prefer not to answer | Do not know

5. What is your gender? (Circle **ALL** that apply)

Female | Male | Trans – Female to Male | Trans – Male to Female

Intersex | Prefer not to answer | Do not know | Other

6. What is your sexual orientation? Choose **ONE**.

- ☐ Heterosexual ("Straight", male/female relationships)
- ☐ Gay
- ☐ Lesbian
- ☐ Bisexual
- ☐ Two-Spirit
- ☐ Queer
- ☐ Prefer not to answer
- ☐ Do not know
- ☐ Other

7. What was your total family income before taxes last year? Choose **ONE**.

- ☐ $0 to $29,999
- ☐ $30,000 to $59,999
- ☐ $60,000 to $89,999
- ☐ $90,000 to $119,999
- ☐ $120,000 to $149,999
- ☐ $150,000 or more
- ☐ Prefer not to answer
- ☐ Do not know

8. How many people does your income support? Choose **ONE**.

- ☐ 0
- ☐ 1
- ☐ 2
- ☐ 3
- ☐ 4
- ☐ 5
- ☐ 6
- ☐ 7
- ☐ 8
- ☐ 9
- ☐ 10+
- ☐ Prefer not to answer
- ☐ Do not know

9. In what language would you prefer to read healthcare information? Choose **ONE**.

English	Hindi	Slovak	Do not know
Amharic	Hungarian	Somali	
Arabic	Inuktitut	Spanish	
Bengali	Italian	Serbian	
Braille	Karen	Tagalog	
Chinese (Simplified)	Korean	Tamil	
Chinese (Traditional)	Nepali	Tigrinya	
Cree	Ojibwe	Turkish	
Czech	Oji-Cree	Twi	
Dari	Polish	Ukrainian	
Farsi	Portuguese	Urdu	
French	Punjabi	Vietnamese	
Greek	Russian	Other	
Hebrew	Serbian	Prefer not to answer	

10. What is your religious or spiritual affiliation? Check **ONE** only.

- ☐ I do not have an religious or spiritual affiliation
- ☐ Christian Orthodox
- ☐ Protestant
- ☐ Roman Catholic
- ☐ Christian, not included elsewhere on this list
- ☐ Animism or Shamanism
- ☐ Atheism
- ☐ Baha'i Faith
- ☐ Buddhism
- ☐ Confucianism
- ☐ Hinduism
- ☐ Islam
- ☐ Jainism
- ☐ Jehovah's Witness
- ☐ Judaism
- ☐ Native Spirituality
- ☐ Pagan
- ☐ Rastafarianism
- ☐ Sikhism
- ☐ Spiritualism
- ☐ Unitarianism
- ☐ Zoroastrianism
- ☐ Other
- ☐ Prefer not to answer
- ☐ Do not know

11. What type of housing do you live in? Choose **<u>ONE.</u>**

- ☐ Own Home
- ☐ Renting Home
- ☐ Boarding Home
- ☐ Correctional Facility
- ☐ Homeless/on Street
- ☐ Group Home
- ☐ Shelter/Hostel
- ☐ Supportive Housing
- ☐ Other
- ☐ Prefer not to answer
- ☐ Do not know

Thank you for participating in this survey.
